# Clinical evidence based review and systematic scientific review in the identification of malignant transformation of inverted papilloma

**DOI:** 10.1186/s40463-020-00420-x

**Published:** 2020-04-30

**Authors:** Cai Long, Basel Jabarin, Alexandra Harvey, Jennifer Ham, Amin Javer, Arif Janjua, Andrew Thamboo

**Affiliations:** 1 Faculty of Medicine, University of British Columbia, Vancouver, Canada; 2 St Paul’s Sinus Centre, University of British Columbia, Vancouver, BC Canada

**Keywords:** Inverted papilloma, Sinonasal inverted papilloma, Malignancy transformation, Squamous cell carcinoma, Schniederian papilloma, Clinical surveillance, Evidence based review

## Abstract

**Background:**

Inverted papilloma (IP) is an unusual type of benign tumor that has high recurrence rates and the potential to transform into squamous cell carcinomas (SCC). The mechanism of the transformation process from IP to IP-SCC is uncertain and there is no consensus regarding the best practice for IP-SCC detection. The goal of this study is to identify the best clinical methods to detect for IP-SCC.

**Methods:**

An evidence-based review was performed using Medline and Ovid to obtain all articles up to October 10th, 2019 pertaining to identification of IP malignant transformation. All manuscripts discussing clinical methods or biomarkers were included.

**Results:**

Based on clinical research studies, convoluted cerebriform pattern and apparent diffusion coefficient values on Magnetic Resonance Imaging (MRI) can help differentiate benign IP from SCC and increased SUVmax on PET/CT is associated with higher probability of malignancy although not as specific. No consensus about the best biomarker for IP-SCC has been reached among researchers and continues to be exploratory.

**Conclusion:**

Endoscopy with biopsy is the gold standard practice to identify IP-SCC; however, MRI is the preferred imaging modality to recognize malignant transformation in cases where biopsy is difficult. Multiple biomarkers have shown positive results, but no single indicator with clinical significance for monitoring malignant transformation process has been found.

## Background

Inverting papilloma (IP) is a challenging condition to manage in the sinonasal cavity. A recent literature review has shown that the best surgical approach to the management of IP is endoscopic [1]. However, the challenge remains on how to best surveillance these patients postoperatively, especially if the recurrence converts to malignant transformation. To our knowledge, the most up to date IP recurrence rate is 13.72% with a malignant transformation potential of 7.6% [1–3]. Given that the recurrence and malignant transformation can happen more than 8 years after the preliminary surgery, follow-up of 3 years or greater is recommended [4, 5].

The reported incidence of malignancy in IP varies greatly in the literature, and has been reported as high as 27%, but most commonly is seen to be between 5 and 10% [2, 6–8]. The majority of the malignant cases are squamous cell carcinoma (SCC), it can form synchronously or metachronous from IP. In a meta-analysis in 2007, Mirza et al. found a risk of 7.1% of synchronous carcinomas and a risk of 3.6% of metachronous malignancies [8]. Re et al. identified a 9% overall rate of malignant transformation in 2017 [2]. No significant correlation was found between tumor stage and histologic differentiation in these IP-associated malignancies [9]. The survival rates for patients with malignant transformation are quite low, with the five-year post-surgery survival rate ranging from 39.6 to 65.7% and an average survival of 3.4 years [10, 11].

While the most common surveillance technique is endoscopy [1] followed by a biopsy if there is a concerning finding, surgeons struggle to check areas in the nose that cannot be easily visualized such as the lateral frontal sinus, the anterior medial maxillary sinus and sinus cavities that have stenosed due to post-surgical healing. Therefore, surgeons require other surveillance techniques to best identify incidents of IP recurrence but more importantly the transformation to SCC. Moreover, it would be beneficial to know a priori which IPs have a higher risk of transformation. There is growing understanding regarding the biology of IP transforming to SCC; it is therefore important to summarize this body of literature as it may provide surgeons with another way of determining whom to follow up more closely. Recommendations will be made based on levels of evidence while weighing benefits and risks. As recommendations may not apply to all IP patients, clinical judgement is required on a per case basis.

## Methods

This article was written following the evidence-based-review method established by Rudmik et al. [12] A systematic review of the literature was conducted on Medline and Ovid up to October 10, 2019 by combining “sinonasal inverted papilloma.mp or papilloma, inverted/ or Schniederian papilloma” and “Carcinoma, Squamous Cell/ or malignant.mp or malignancy.mp”. After removing duplicates, 363 papers were found, and 7 surveillance options to identify the malignant transformation were identified (Table 1). Another literature search for each individual surveillance option was then performed using the key words described above. All reviewed abstracts were required to have a clearly defined goal of identify the malignant transformation process (IP-SCC).

**Table 1:** Surveillance options for IP malignant transformation

Clinical investigation options	Endoscopy
	Computed Tomography
	Magnetic Resonance Imaging
	PET-CT with FDG
Biologic markers	Genetic factors (P53, P21, MSX2, PDCD4, KRAS, PTEN)
	Proteins (SCCa, fascin, surviving, COX-2, cell adhesion molecules, Ki-67 etc)
	Viral (HPV, EBV)

The manuscripts of all included studies were reviewed. For clinical surveillance techniques, the level of evidence for each paper was given, followed by a grading system (Table 2) based on the American Academy of Pediatrics Steering Committee on Quality Improvement and Management [13]. For biological markers, a scientific systematic review was performed. Three authors (C.L., J.B. and A.H.) reviewed the literature and wrote the initial manuscripts. The paper and the literature were then reviewed in detail by the senior author (A.T.). Subsequent authors (A.J. and A.J) reviewed the manuscript and critically appraised the paper following the online iterative process set by Rudmik and Smith [12]. Final recommendations were based on quality of research and balance of benefit vs. harm.

**Table 2:** Defined grades of evidence and recommendations [AAP ref]

Grade	Research	Preponderance of benefit over harm	Balance of benefit and harm
A	Well designed RCTs or diagnostic studies	Strong recommendation	Option
B	RCT or diagnostic studies with minor limitations; overwhelming consistent evidence from observational studies	Strong recommendation / recommendation	Option
C	Observation studies (case control and cohort design)	Recommendation	Option
D	Expert opinion; case reports; reasoning from first principles	Option	No recommendation

## Results

After reviewing 249 papers, the results were summarized together under ‘clinical surveillance techniques’, ‘computed tomography’, ‘magnetic resonance imaging’, ‘PET-CT with FDG’, and ‘biological markers’.

### Clinical surveillance techniques

#### General principles

Certain general demographics might influence how clinicians schedule surveillance plans for patients. Therefore, in order to guide clinical decisions, All studies that discussed the location, follow-up and clinical risk factors associated with IP-SCC were included in this review.

##### Location (Table 3)

Most IP-SCC originated from the ethmoid region, maxillary sinus and lateral nasal wall [11]; however, there were multiple cases reporting IP-SCC in the middle ear, oral cavity, lacrimal sac and pituitary fossa [15–20, 22]. A complete head and neck examination is recommended to follow up patients with IP, as IP can reoccur and transform into malignancy in the primary site, or in an alternate location.

**Table 3:** General location for IP malignant transformation surveillance

Study authors	Year	Study design	Level of evidence	Study groups	Intervention	Outcome	Conclusion
Lin et al. [14]	2014	Observational study	C	129 patients, 19 SCC (14.7%)	Medical history reviewed.	IP requires follow up of more than 2 years. 5 year survival for IP with SCC is 61%. No clear predictors of malignancy were seen in this study.	Smoking impacts carcinomatous progression. The most common lesion location was the medial maxillary sinus, maxillary sinus and ethmoid sinus. The interval between initial resection and diagnosis of malignancy was 73 months.
Mazlina et al. [15]	2006	Case report	D	1	N/A	N/A	A case with a multicentric IP in the sinonasal region and middle ear of a 54 year old man.
Bernat et al. [16]	1998	Case report	D	1	N/A	N/A	A case where an epidermoid carcinoma developed from the inverted papilloma.
Dingle et al. [17]	2012	Case report	D	1	N/A	N/A	The first case of bilateral IP of the middle ear with intracranial involvement and malignant transformation.
Mathew et al. [18]	2012	Case report	D	1	N/A	N/A	A case of SIP (malignant) with neck metastasis.
Islam et al. [19]	2014	Case report	D	1	N/A	N/A	A case with IP transformation to SCC of the lacrimal sac, bilateral, and development of distant metastasis.
Sharma et al. [20]	2015	Case study	D	1	N/A	N/A	Follow up should include complete head and neck examination for patients with a typical SIP, as SIP can be re-occur in many locations.
Balasubramani et al. [21]	2009	Case study	D	1	N/A	N/A	IP-SCC can invade into the pituitary fossa mimicking a pituitary macroadenoma.
Garcia et al. [22]	2014	Case study	D	1	N/A	N/A	A case of IP-SCC arise from maxillary sinus extending to the mouth

##### Follow-up (Table 4)

IP requires follow-up of more than 3 years, due to the high rate of delayed recurrences [7]. The follow up duration should be based on the interval between initial resection and malignancy diagnosis of recurrent IP-SCC. The mean interval varies from 34.4 months to 40 months [3, 10]. One study by Lin et al. in 2014 showed that the mean interval was on average 73 months [14].

**Table 4:** Follow up duration of IP malignant transformation surveillance

Study authors	Year	Study design	Level of evidence	Study groups	Intervention	Outcome	Conclusion
Lin et al. [14]	2014	Observational study	C	129 patients, 19 SCC (14.7%)	Medical history reviewed.	IP requires follow up of more than 2 years. 5 year survival for IP with SCC is 61%. No clear predictors of malignancy were seen in this study.	Smoking significantly affected overall survival by impacting carcinomatous progression. The most common lesion location was the medial maxillary sinus, maxillary sinus and ethmoid sinus. The mean interval between initial resection and diagnosis of malignancy was 73 months.
Liang et al. [10]	2015	Observational study	C	213 patients, 87 SCC.	Medical history reviewed.	Age, synchronous or metachronous tumors, and pathological stage were independent risk factors for mortality.	Factors associated with significantly poor prognosis were: advanced-stage, metachronous tumors, and cranial base or orbit invasion. Mean follow up time was 40 months.
Nudell et al. [3]	2014	Observational study	C	20 IP-SCC	Medical history reviewed	An average of 34.4 months between first diagnosis of SP to IP-SCC identified.	SP that may undergo malignant transformation are nearly impossible to identify based on morphologic examination.

##### Clinical risk factors (Table 5)

It is reasonable to frequently follow up with patients who are at a higher risk of malignancy. Unfortunately, there is not enough high-level evidence about the risk factors leading to malignant transformation. In a grade C study, it was found that factors associated with significantly poor prognosis were advanced-stage, metachronous tumors, and cranial base or orbital invasion [10]. Several studies show that female sex and smoking were related to recurrence of IP but no studies showed whether these factors would lead to greater incidence of IP-SCC transformation. Many researchers tried to retrospectively study whether factors such as age, clinical stage or industrial occupations affects the prognosis of IP, often reaching contradictory results [24, 25]. There is no consensus regarding whether clinical stage, treatment method, age, or occupation predict the malignant transformation of IP. Gender and history of sinus disease are found to be irrelevant to prognosis [23]. Controversies exist with regards to whether smoking increases the risk of malignant transformation of IP [14, 26, 27]. Some studies have attempted to differentiate IP from IP-SCC using clinical features such as epistaxis, pain, and bone destruction, but these are usually quite non-specific and inconsistent [28].

**Table 5:** Clinical risk factors of IP malignant transformation

Study authors	Year	Study design	Level of evidence	Study groups	Intervention	Outcome	Conclusion
Yu et al. [23]	2014	Observational study	C	356 IP patients, 32 transformed to malignant tumor (21 SCC).	Cases of patients were followed up from 23 to 212 months.	5-year survival rate is 72.5%, median survival time was 62.2 months. 8.99% of all SNIP cases transformed into SCC, the incidence of malignancy among SNIP cases was 11%. Male to female malignant ratio: 3.6:1	The main factors affecting prognosis were clinical stage and treatment method. Gender and age of onset are irrelevant to prognosis. Lower staging increase life expectancy. Invasion of orbit and skull predict poor prognosis.
Kim et al. [7]	2012	Observational study	C	578 pts., 22 (3.8%) SCC.	578 IP patients from 17 hospitals included for recurrence analysis. Mean follow-up - 41 months.	15.7% had recurrences. Patients whose IPs involved the frontal sinus or the medial wall of the maxillary sinus had higher recurrence rates. 136 originated from multiple sites.	There was no significant difference in recurrence rates based on stage or surgical approach. Given the rate of delayed recurrence, follow-up of >3 years required. Rcurrences are higher for IP with an original site of frontal sinus or medial wall of MS.
Sham et al. [24]	2010	Observational study	C	50 pts. vs. 150 matched controls	IP and control group patients were interviewed and data analyzed.	Outdoor and industrial occupations (driver, construction worker) were associated with IP.	Tobacco smoking, drinking alcohol, history of allergic rhinitis, sinusitis, nasal polyp, non-sinonasal papilloma and non-sinonasal malignancy were not significant factors.
Jardine et al. [25]	2000	Observational study	C	89 pts., 2 SCC	IP patients’ medical histories reviewed.	Mean follow up 2.1 years.	Younger patients were more likely to recur. Smokers tend to have multiple recurrence. P53 is unlikely to be of help in predicting the clinical behavior of IP. Dust exposure is unrelated to recurrent disease.
Hong, Sung-Lyong [26]	2013	Observational study	C	162 IP patients, 17 SCC (9 synchronous, 3 metachronous)	IP patients; medical histories reviewed.	Recurrence rate is 28.6%, mean period of recurrence was 6.3 months.	Smoking history increased the risk of malignant transformation of SIP (odds ratio: 12.7).
Il Joon Moon [27]	2010	Observational study	C	132 IP patients, 9 SCC	IP patients; medical histories reviewed.	No association between smoking history and synchronous malignancy was found.	There was a strong association between staging and malignant transformation. Smoking increases recurrence.

#### Endoscopy

Clinical examination, particularly endoscopic exams, are crucial for surveillance. Currently the confirmation diagnosis of IP-SCC depends on histopathological results. Clinicians use endoscopic sinonasal examination to monitor for a recurrence of an IP. However, it is often difficult to detect a malignant transformation of a benign IP from visual inspection, particularly if the tumors are not easily visible by nasal endoscopy. Moreover, IP-SCC often do not involve the entire tumor, hence the biopsies can be non-representative and are not highly reliable [9].

- Aggregate grade of evidence: NA
- Benefit: direct visual inspection, easily accessible, biopsy is the gold standard of confirming presence of IP-SCC.
- Harm: bleeding and infection risk, chance of missing the tumor.
- Cost: low
- Benefit harm assessment: Benefit outweighs harm.
- Value judgement: Endoscopy followed by a biopsy is the first line method for IP-SCC identification.
- Recommendation level: Recommended.
- Intervention: First line method for long term surveillance.

#### Computed tomography

There are no studies that support the use of CT to identify the presence of IP-SCC (Table 6). A level 4 study by Chawla et al. indicated that IP on a CT scan can show bone remodeling and resorption rather than osseous destruction, which is more often seen in malignant tumors [29]. Miyazaki et al. found that bone destruction was significantly higher in IP-SCC with 3 out of 5 IP-SCC patients were found to have bone destruction [28]. The finding was quite nonspecific. The studies do not prove the efficacy of CT scans in differentiating IP from IP-SCC.

- Aggregate grade of evidence: NA (only 1 Level 4 study)
- Benefit: No evidence differentiating IP from IP-SCC on CT scan.
- Harm: Concerns regarding radiation exposure.
- Cost: Moderate
- Benefit harm assessment: No benefit for IP-SCC identification.
- Value judgement: Require more research
- Recommendation level: No recommendation
- Intervention: None.

**Table 6:** Computed Tomography in surveillance of IP malignant transformation summary

Study authors	Year	Study design	Level of evidence	Study group	Intervention	Outcome	Conclusion
Chawla et al. [29]	2016	Expert opinion	D	None	None	IP on CT appears as lobulated soft tissue density mass with or without any calcification. IP causes bone remodeling and resorption rather than osseous destruction (as seen in malignant tumors).	CCP on MRI combined with osseous remodeling on CT are highly suggestive of IP over malignant mass.
Miyazaki et al [28]	2018	Observational study	C	70 IP cases, 6 IP-SCC	Medical history reviewed, comparing with histological findings	Nasal bleeding, pain, bone destruction and extent of disease on CT and MRI are associated with malignancy.	Imaging findings are associated with IP-SCC.

#### Magnetic resonance imaging

Five studies were included in the review (Table 7). Convoluted cerebriform pattern (CCP), alternating hypointense and hyperintense bands on T2 weighted and contrast enhanced T1 weighted images have been reported as a reliable MRI features of IPs [30]. CCP has been found in all IP cases as well as in some malignant tumors [29, 32]. In IP, CCP is generally diffuse. A focal loss of CCP pattern maybe indicative of a malignancy [35]. A recent study showed that loss of CCP and low apparent diffusion coefficient (ADC) values on MRI with diffusion-weighted imaging can help differentiate IP-SCC from IP with 100% specificity (grade B evidence) [30]. Bone destruction with or without sinonasal extension could be used as an indication of IP-SCC(grade C evidence) [34]. Because IP causes bone remodeling and resorption rather than osseous destruction, Zhang et al. suggested that diffused CCP on MRI combined with bone remodeling on CT may be highly suggestive of IP over IP-SCC [36].

- Aggregate grade of evidence: Level 2: 2 studies; Level 3: 3 studies;
- Benefit: MRI imaging feature can help differentiate IP from IP-SCC.
- Harm: Repeated exposure to gadolinium, claustrophobia, and heating/dislodgement of implanted metal.
- Cost: Moderate/High.
- Benefit harm assessment: Benefit outweighs harm.
- Value judgement: Require more research.
- Recommendation level: Recommended.
- Intervention: Using CCP and ADC value, MRI has capacity to identify IP-SCC. MRI is recommended if biopsy is not achievable or there are concerns of the accessible part of the tumor to biopsy inaccurately representing the entirety of the tumor.

**Table 7:** MRI in surveillance of IP malignant transformation summary

Study authors	Year	Study design	Level of evidence	Study group	Intervention	Outcome	Conclusion
Yan et al. [30]	2018	Diagnostic study	B	76 IP patients, 66 IP-SCC patients	MRI results were compared with surgical pathology reports.	IP have higher prevalence of CCP on MRI, and higher ADC values.	Evaluation of CCP and ADC values on MRI can help differentiate benign IP from IPSCC.
Oikawa et al. [31]	2010	Diagnostic study	B	21 IP patients	Patient were staged based on MRI findings and the results were compared with pathological findings..	The positive predictive value of MRI staging, as verified by surgical and pathological findings, was 68 to 89%.	MRI can be used to accurately predict the extent of tumor involvement and staging.
Jeon et al. [32]	2008	Observational study	C	30 IP pts., 8 with SCC, vs 128 patients with various other malignant sinonasal tumors	MR images of two groups of patients were reviewed.	CCP was found in all IPs and some of the malignant sinonasal tumors. Of patients who had IP with coexistent SCC, 4 had focal loss.	The sensitivity and specificity of using CCP to differentiate IP from other malignant tumors are 100 and 87%, respectively. CCP pattern cannot be used to discriminate IP from IP with SCC.
Maroldi et al. [33]	2003	Observational study	C	23 IP patients vs. 23 malignant tumor (9 SCC)	MR images of IP and malignant patients are reviewed and compared.	CCP are found in all IP cases by SE T1 images, and in only 1 of MTs.	A columnar pattern (CCP) is a reliable MRI indicator of IP, and reflects its histological architecture, CCP and bone erosion status can distinguish IP from malignancies.
Wang et al. [34]	2014	Observational study	C	43 IP vs. 45 malignant tumor in nasal cavity (7 SCC)	MR images of IP and malignant patients are reviewed and compared.	There were significant differences between IP and malignant tumors. Washout-type TIC had a higher sensitivity and specificity in diagnosis of malignant tumors in the nasal cavity.	Non-enhanced and static MRI combined with dynamic contrast enhanced MRI (DCE-MRI) could improve differentiation between IP and malignant tumors.

#### Positron-emission tomography with 18F-fluoro-deoxyglucose (PET-CT with FDG)

PET-CT studies have shown that IP patients have higher SUV max than non IP patients, and IP-SCC patients have higher SUVs than IP patients (Table 8) [37]. However, Joen et al. found that SUVs for IPs can at times be high leading to a false diagnosis of IP-SCC [35]. Allegra et al. reported a case where PET-CT failed to identify the primary malignant site because there was no SUV increase in the IP-SCC site [38]. Consequently, PET-CT with FDG can be used as an adjunct tool but cannot be solely relied upon making the diagnosis of IP-SCC.

- Aggregate grade of evidence: Level 2: 2 studies; Level 3: 2 studies; Level 4: 2 studies.
- Benefit: SUV max can increase suspicion of IP-SCC.
- Harm: Radiation exposure.
- Cost: High.
- Benefit harm assessment: Equal balance of benefit to harm.
- Value judgement: Challenging to recommend PET-CT use in the identification of IP-SCC due to contradicted research results and costs.
- Recommendation level: None.
- Intervention: None.

**Table 8:** PET/CT in surveillance of IP malignant transformation summary

Study authors	Year	Study design	Level of evidence	Study groups	Intervention	Outcome	Conclusion
Jeon et al. [35]	2009	Diagnostic study	B	8 IP patients, 6 of them with IP associated SCC.	SUVs of PET/CT images and CCP of MR images are reviewed and compared. SCC are confirmed by histologic exam.	In PET/CT study, IP with SCC has consistently higher SUVs than IPs without SCC; however this test has low specificity. MRI findings showed wide discrepancy in terms of CCP distribution. Aggressive bone destruction was found in most SCC patients.	PET/CT cannot be used reliably to predict malignancy yet due to limited data. Focal loss of CCP on MR might not be additional sign of malignancy.
Allegra et al. [38]	2012	Diagnostic study	B	12 cases (7 IP)	18 FDG - PET/CT of IP patients were analyzed and compared with histological results.	For IP patients the SUVmax value is larger than for non IP patients.	Lesions with a negative or diffuse 18FDG uptake with SUVmax less than 3 should be considered negative for IP.
Shojaku et al. [85]	2007	Observational study	C	5 IP patients, 2 of them have IP associated SCC	FDG PET was performed on IP patients and SUVmax was analyzed.	High FDG uptake (SUVmax) was observed in all patients, with a higher SUVmax in SCC patients.	The SUVmax of IP can warn the physician of the probability of an associated malignancy.
Yilmaz et al. [37]	2015	Observational study	C	8 nasal polyps vs 10 IP vs 9 SCC	PET CT of 27 patients were analyzed.	The mean SUVmax was found to be high in the IP group, and highest in the SCC group.	High SUVmax can be used to rule out nasal polyp. The SCC group had a higher SUVmax.
Zhang et al. [36]	2015	Case report	D	1 IP patient	SIP with co-existent malignancy and cervical metastasis was reviewed.	PET failed to identify the primary malignancy site because there was no SUV increase in SCC.	FDG PET/CT may be not a reliable predictor of malignancy in SIP.
Kim et al. [86]	2017	Case report	D	1 IP patient	Patient had two operations and 2 sequential PET CT scans.	N/A	The SUV of IP could vary over time in PET CT. PET CT is not an ideal tool to distinguish IP from other inflammatory polyps or cancer.

### Biological markers

#### Genetic factors

Multiple genetic factors have been identified as potential malignant transformation markers for IP.

P53 is a transcription factor controlling cellular proliferation and apoptosis via the regulation of genes involved in cell-cycle arrest, DNA repair, and apoptosis [39]. Ten retrospective studies investigating the association between P53 levels and IP malignant transformation were found,. Four studies support a positive association of mutant P53 level and malignant transformation [40–43], three studies reported no relation between level of mutant P53 and transformation rate [39, 44, 45] and the remaining studies were inconclusive [46, 47]. No conclusion can be drawn about whether P53 is involved in the malignant transformation process.

P21 cyclin-dependent kinase inhibitor is a cell cycle progression control factor. It promotes cell cycle arrest in response to a variety of stimuli. The inhibitory effect of P21 on cell cycle progression correlates with its nuclear localization. P21 can be induced by both p53-dependent and p53-independent mechanisms [40, 41]. Two out of four reviews supported P21 to play a role in malignant transformation and to be a pathological marker for malignant transformation [42, 46], and two studies reported no association of P21 levels with malignant transformation [39, 47]. Both used immunohistochemical staining to make retrospective comparisons of pathological specimens from IP, IP with dysplasia, and SCC.

Muscle segment homeobox gene MSX2 is implicated in cellular differentiation and survival. Zhang et al. [48] noted significantly higher levels of MSX2 mRNA in IP-SCC than in controls and Wu et al. observed expression of MSX2 in 100% of IP-SCC and significantly greater expression in IP with severe dysplasia than in IP and control [49]. These studies support the potential use of MSX2 as a target for monitoring malignant transformation.

PDCD4 is a tumor suppressor gene that has been previously implicated in the progression of many types of tumours. Compared to controls, Wang et al. noted a significant decrease in PDCD4 expression in IP [50]. Furthermore, altered expression of the gene was correlated with dysplastic features, indicating that it may be used to predict IP progression and malignant transformation.

Targeted amplicon sequencing by Yasukawa et al. identified significant genetic mutation of the KRAS gene in SCC-associated and dysplastic IP as compared with nondysplastic IP [51]. The use of KRAS mutation to predict malignant transformation had a sensitivity of 85%, a specificity of 90%, a positive predictive value 91% and a negative predictive value of 75%, indicating its effectiveness as a marker.

Phosphatase and tensin homologue, PTEN, is a tumor suppressor gene. Zhang et al. found that decreased expression of PTEN was associated with both IP dysplasia and malignant transformation [52]. While Kakizaki et al. found no significant difference in expression between non-dysplastic and dysplastic IPs, they similarly noted decreased PTEN in SCCs as compared to IPs, validating its use as a biomarker of carcinogenesis [52, 53].

#### Proteins

Squamous cell carcinoma antigen (SCCa) is one of the most reliable tumor markers for squamous cell carcinoma. Three studies associated a high level of serum SCCa with IP progression, growth, and recurrence [54–56]. This might help the surgeon in the postoperative setting by identifying high-risk patients and planning follow-up strategy tailored to the individual patient.

Fascin is an actin cross-link binding protein required for the formation of actin-based cell-surface protrusions and cell motility. Fascin up-regulation in lung, gastric, breast and hepatobiliary carcinomas correlates with aggressiveness and decreased survival. Two retrospective studies examined the association between fascin and IP-SCC by using immunohistochemical staining of specimens taken from different groups: IP, IP with high dysplasia, IP with SCC and normal control tissue. It was found that fascin expression increased gradually and significantly with the progression and severity of dysplasia [57–59].

Survivin, an inhibitor of apoptosis, is also found to be potentially correlated with SCC. Nuclear survivin expression was significantly higher in SCCs than in IPs in one study [60].

Two studies found that increased expression of COX-2, a rate-limiting enzyme in prostaglandin synthesis, might be linked with malignant transformation [51, 61, 62]. The osteopontin-VEGF (vascular endothelial growth factor) axis has been implicated in tumor progression and angiogenesis. Liu et al. and Wu et al. found significantly greater immunohistochemical staining and mRNA expression of OPN and VEGF in higher-stage IP specimens than in lower-stage tissues [63, 64].

E-cadherin and β-catenin are cell adhesion molecules commonly used to mark the epithelial to mesenchymal transition [65]. Correspondingly, an immunohistochemical analysis by Koo et al. demonstrated lower levels of E-cadherin and β-catenin in IP-SCC than in IPs [66], and Stasikowska-Kanicka et al. found the immunoexpression of E-cadherin to be decreased in SCC as compared to IP [65].

Ki-67 is a marker of cell proliferation, in immunohistochemical studies of protein expression, Tsou et al. observed significantly greater Ki-67 staining in IP-SCC as compared with non-recurrent IP [67]. Ki-67 was considered a signal of poor prognosis and malignant transformation [67].

Sahnane et al. identified that occupation exposure increases LINE-1 hypomethylation which might be an epigenetic marker [68].

### Viral

Because IP shares certain clinical characteristics with recurrent respiratory papillomatosis, namely high recurrence, confined aggressiveness, and malignant transformation potential, Jalilvand et al. contend that IP likely also has an infectious etiology [69]. Identifying such an agent would prove invaluable in predicting tumor progression and developing future therapies. Human papillomavirus (HPV) has long been suggested and investigated as a potential driving agent. HPV is a DNA virus that shows tropism for epithelium, leading to epidermal and mucosal infections wherein the virus is integrated into the host DNA.

In IP specimens, HPV positivity ranged between 9 and 60%. Comparatively, controls ranged between 0 and 7.6% [7, 69–77]. The most common HPV strains observed in IP were HPV-6 and HPV-11. Significantly higher HPV positivity was found in cases with moderate to severe dysplasia as compared cases with low grade dysplasia [74, 78]. There is good evidence that HPV infection is implicated in the tumorigenesis of IP. Regarding malignant transformation, HPV positivity in SCC tissues has been recorded as between 0 and 100% [69–73, 75–77, 79]. Inconsistencies in reported numbers have been attributed to small sample sizes, degradation of DNA in paraffin-embedded tissues, use of inferior techniques of detection, and geographic factors. Udager et al. found that EGFR mutations and HPV infection may represent alternative oncogenic mechanisms for IPSCC [80]. The most common HPV strains observed in SCC were the high-risk strains HPV-16 and HPV-18. SCCs have been associated with significantly higher viral loads than IPs and controls. Yamashita et al. found in a study that all 5 SCC and 16 IP-SCC patients with HPV-16 infection showed mixed type integration, whereas the majority of IPs and controls have demonstrated HPV in its benign episomal form [72, 77]. Rooper et al. reported that transcriptionally active high-risk HPV does not play a common role in its malignant transformation into IP-SCC based on a study of 59 tumors [81].

Although the relationship of HPV infection to malignant transformation remains somewhat controversial [68, 82], a recent meta-analysis by Zhao et al. found a close association between HPV infection and malignancy, with a pooled odds ratio of 2.16 [83]. Furthermore, high-risk subtypes HPV-16, HPV-18, and HPV-16/18 coinfection had even stronger correlations, with odds ratios of 8.8, 8.04, and 18.57 respectively. In order to rule out incidental infection due to increased susceptibility of malignant tissues, further studies elucidating the molecular mechanisms of anti-apoptotic derangements and tumorigenesis are required [84].

## Discussion

Using evidence-based and systematic scientific review methods, this study aimed to elucidate the usefulness of numerous clinical investigative options for characterising IP-SCC. IP-SCC can either be found at the initial presentation, or during follow-ups when recurrences are identified. The literature does not provide findings that differentiate between the two scenarios. The senior authors recommend that the endoscopic exam followed by a biopsy be the gold standard in identifying IP-SCC. However, there are clinical scenarios where the endoscopic exam may not be possible, or concern that the biopsies are non-representative. We recommend clinicians follow patients with MRI. Given the high recurrence rate in the first 2 years following the original resection, and the mean interval between first resection and malignancy is about 40 months, our clinical practice is to do serial MRI scans for 3 years and then a repeat MRI at 5 years when the endoscopic exam may not be possible, or concern that the biopsies are non-representative. Also, in these scenarios, a complete head and neck examination is recommended on a yearly basis due to the sporadic sites of IP-SCC can be found.

The senior authors do not use CT in elucidating the presence of IP-SCC due to lack of strong evidence. While there is evidence to support the use of PET-CT, the senior authors do not use PET-CT to determine the presence of IP-SCC due to costs and access. If the MRI is suspicious, the authors will take the patient to the operating room to get a frozen section biopsy. If it is positive for SCC, a PET-CT is done post-operatively to determine if there is metastatic spread of the tumor.

The future of surveillance will include biological markers. While this study clearly illustrates there is no utility in using biomarkers at the present time, there are several biological markers that have shown great potential as prognostic indicators. Genetic factors such as MSX2, PDCD4, KRAS, PTEN are thought to play an important role in malignant transformation. Several proteins, including SCCa, fascin, survivin, COX-2, cell adhesion molecules and Ki-67 are found in higher levels in IP-SCC than in IP. Further research in this area may allow for an accessible test for clinicians to use to help risk stratify IP patients.

## Conclusion

Based on the evidence level of various methods to identify IP-SCC, while histological diagnosis is the gold standard for the diagnosis of malignant transformation, clinicians should keep in mind of the sampling error of endoscopic biopsy. MRI as a standalone technique is the preferred modality in recognizing malignant transformation of inverting papilloma. Further clinical trials are required to reinforce these findings. This study also identified a number of biological markers that may become key markers in the future for identifying patients that require close follow-up.

## Data Availability

Data sharing is not applicable to this article as no datasets were generated or analysed during the current study.

## Abbreviations

IP: Inverted papilloma
SCC: Squamous cell carcinoma
MRI: Magnetic resonance imaging
CT: Computed tomography
PET-CT with FDG: Positron-emission tomography with 18F-fluoro-deoxyglucose
CCP: Convoluted cerebriform pattern
ADC: Apparent diffusion coefficient
SCCa: Squamous cell carcinoma antigen
HPV: Human papillomavirus

## References

[1] Peng R, Thamboo A, Choby G, Ma Y, Zhou B, Hwang PH. Outcomes of sinonasal inverted papilloma resection by surgical approach: an updated systematic review and meta-analysis. Int Forum Allergy Rhinol. 2019. 10.1002/alr.22305. 10.1002/alr.22305 30748098

[2] Re M, Gioacchini FM, Bajraktari A (2017). Malignant transformation of sinonasal inverted papilloma and related genetic alterations: a systematic review. Eur Arch Oto-Rhino-Laryngol..

[3] Nudell J, Chiosea S, Thompson LDR (2014). Carcinoma ex-Schneiderian papilloma (malignant transformation): a Clinicopathologic and Immunophenotypic study of 20 cases combined with a comprehensive review of the literature. Head Neck Pathol.

[4] Myers EN, Fernau JL, Johnson JT, Tabet JC, Barnes EL. Management of Inverted Papilloma. Laryngoscope. 1990;100(5):481???490. doi:10.1288/00005537-199005000-00008. 10.1288/00005537-199005000-00008 2184303

[5] Kleihues P, Sobin LH (2000). World Health Organization classification of tumors. Cancer..

[6] Podd TJ, Dawes PKDJ, Marshall HF (1994). Malignant inverted papillomas: a review of seven cases and implications for treatment. Clin Oncol.

[7] Kim D-Y, Hong S-L, Lee CH (2012). Inverted papilloma of the nasal cavity and paranasal sinuses: a Korean multicenter study. Laryngoscope..

[8] Mirza S, Bradley PJ, Acharya A, Stacey M, Jones NS (2007). Sinonasal inverted papillomas: recurrence, and synchronous and metachronous malignancy. J Laryngol Otol.

[9] Choi JW, Kim SG, Kim Y-M, Yoon Y-H, Kim AY, Rha K-S (2012). Clinical and histologic features of inverted papilloma–associated malignancy. Eur Arch Oto-Rhino-Laryngol.

[10] Liang QZ, Li DZ, Wang XL, Huang H, Xu ZG, Wu YH (2015). Survival outcome of squamous cell carcinoma arising from sinonasal inverted papilloma. Chin Med J.

[11] Lobo BC, D’Anza B, Farlow JL (2017). Outcomes of sinonasal squamous cell carcinoma with and without association of inverted papilloma: a multi-institutional analysis. Am J Rhinol Allergy..

[12] Rudmik L, Smith TL (2011). Development of an evidence-based review with recommendations using an online iterative process. Int Forum Allergy Rhinol..

[13] Academy A, Recommendations C, Guidelines CP, Pediatrics OF (2004). Classifying Recommendations for clinical practice Guidelines. Pediatrics..

[14] Lin GC, Akkina S, Chinn S (2014). Sinonasal inverted papilloma: prognostic factors with emphasis on resection margins. J Neurol Surg Part B Skull Base.

[15] Mazlina S, Shiraz MARM, Hazim MYS, Amran AR, Zulkarnaen AN, Muhaizan WMW (2006). Sinonasal inverted papilloma with malignant transformation in the middle ear: a multicentric origin?. J Laryngol Otol.

[16] Bernat Gili A, Morais Pérez D, Dalmau Galofre J, Ayerbe TV (1998). Malignant rhinosinusal inverted papilloma with orbital involvement. An Otorrinolaringol Ibero Am.

[17] Dingle I, Stachiw N, Bartlett A, Lambert P (2012). Bilateral inverted papilloma of the middle ear with intracranial involvement and malignant transformation: first reported case. Laryngoscope..

[18] Mathew P, Idiculla JJ (2012). Malignant sinonasal papilloma with neck metastasis: a rare report and literature review. Int J Oral Maxillofac Surg.

[19] Islam S, Eisenberg RL, Hoffman GR (2014). Malignant transformation of metachronous bilateral Schneiderian inverted papilloma of the lacrimal sac: management considerations and the contentious issue of orbital exenteration. Eur Arch Oto-Rhino-Laryngology..

[20] Sharma J, Goldenberg D, Crist H, McGinn J (2015). Multifocal inverted papillomas in the head and neck. Ear Nose Throat J.

[21] Balasubramani Y, Ellul S, Kam A, McLean C, Malham G (2009). Sinonasal inverted papilloma mimicking a pituitary macroadenoma. J Clin Neurosci.

[22] Garcia AS, Bravo-Calderón DM, Ferreira MP, Oliveira DT (2014). Squamous cell carcinoma arising from inverted Schneiderian papilloma: a case report with Oral involvement. Case Rep Otolaryngol.

[23] H-X YU, LIU G (2014). Malignant transformation of sinonasal inverted papilloma: a retrospective analysis of 32 cases. Oncol Lett.

[24] Sham CL, Lee DLY, van Hasselt CA, Tong MCF. A case-control study of the risk factors associated with sinonasal inverted papilloma. Am J Rhinol Allergy. 24(1):e37–40. 10.2500/ajra.2010.24.3408. 10.2500/ajra.2010.24.3408 20109321

[25] Jardine AH, Davies GR, Birchall MA (2000). Recurrence and malignant degeneration of 89 cases of inverted papilloma diagnosed in a non-tertiary referral population between 1975 and 1995: clinical predictors and p53 studies. Clin Otolaryngol Allied Sci.

[26] Hong S-L, Kim B-H, Lee J-H, Cho K-S, Roh H-J (2013). Smoking and malignancy in sinonasal inverted papilloma. Laryngoscope..

[27] Moon IJ, Lee DY, Suh M-W (2010). Cigarette smoking increases risk of recurrence for sinonasal inverted papilloma. Am J Rhinol Allergy..

[28] Miyazaki T, Haku Y, Yoshizawa A (2018). Clinical features of nasal and sinonasal inverted papilloma associated with malignancy. Auris Nasus Larynx.

[29] Chawla A, Shenoy J, Chokkappan K, Chung R (2016). Imaging features of Sinonasal inverted papilloma: a pictorial review. Curr Probl Diagn Radiol.

[30] Yan CH, Tong CCL, Penta M, et al. Imaging predictors for malignant transformation of inverted papilloma. Laryngoscope. 2018. 10.1002/lary.27582. 10.1002/lary.27582 30515841

[31] Oikawa K, Furuta Y, Oridate N (2010). Preoperative staging of sinonasal inverted papilloma by magnetic resonance imaging. Laryngoscope..

[32] Jeon TY, Kim HJ, Chung SK (2008). Sinonasal inverted papilloma: value of convoluted cerebriform pattern on MR imaging. Am J Neuroradiol.

[33] Maroldi R, Farina D, Palvarini L, Lombardi D, Tomenzoli D, Nicolai P (2003). Magnetic resonance imaging findings of inverted papilloma: differential diagnosis with malignant sinonasal tumors. Am J Rhinol.

[34] Xinyan W, Zhengyu Z, Xiaoli C, Jing L, Junfang X (2014). Value of magnetic resonance imaging including dynamic contrast- enhanced magnetic resonance imaging in differentiation between inverted papilloma and malignant tumors in the nasal cavity. Chin Med J.

[35] Jeon TY, Kim H-J, Choi JY (2009). 18F-FDG PET/CT findings of sinonasal inverted papilloma with or without coexistent malignancy: comparison with MR imaging findings in eight patients. Neuroradiology..

[36] S-C Z, Wei L, S-H Z, Zhao K (2015). Inability of PET/CT to identify a primary sinonasal inverted papilloma with squamous cell carcinoma in a patient with a submandibular lymph node metastasis: a case report. Oncol Lett.

[37] Yilmaz I, Reyhan M, Canpolat T (2015). Positron emission tomography evaluation of sinonasal inverted papilloma and related conditions: a prospective clinical study. Kulak Burun Bogaz Ihtis Derg.

[38] Allegra E, Cristofaro MG, Cascini LG, Lombardo N, Tamburrini O, Garozzo A (2012). 18FDG uptake in sinonasal inverted papilloma detected by positron emission tomography/computed tomography. Sci World J.

[39] Trovato MC, Ruggeri RM, Guzzo E (2017). Expression of P53 and isoforms in bening and malignant lesions of the head and neck. Histol Histopathol.

[40] Keles N, Erdamar B, Kaur A, Deger K (2003). p21, p53, and p27 Kip1 alterations in benign and malignant tumors of sinonasal epithelium. Otolaryngol - Head Neck Surg.

[41] Kim S-G, Lee O-Y, Choi J-W (2011). Pattern of expression of cell cycle-related proteins in malignant transformation of sinonasal inverted papilloma. Am J Rhinol Allergy.

[42] Oncel S, Cosgul T, Calli A, Calli C, Pinar E (2011). Evaluation of p53, p63, p21, p27, ki-67 in paranasal sinus squamous cell carcinoma and inverted papilloma. Indian J Otolaryngol Head Neck Surg.

[43] Fan G-K, Imanaka M, Yang B, Takenaka H (2006). Characteristics of nasal inverted papilloma and its malignant transformation: a study of cell proliferation and programmed cell death. Am J Rhinol.

[44] Fang SY, Yan JJ, Ohyama M (1998). Immunohistochemistry of p53 in sinonasal inverted papilloma and associated squamous cell carcinoma. Am J Rhinol.

[45] Katori H, Nozawat A, Tsukuda M (2006). Relationship between p21 and p53 expression, human papilloma virus infection and malignant transformation in sinonasal-inverted papilloma. Clin Oncol.

[46] Saegusa M, Nitta H, Hashimura M, Okayasu I (1999). Down-regulation of p27Kip1 expression is correlated with increased cell proliferation but not expression of p21waf1 and p53, and human papillomavirus infection in benign and malignant tumours of sinonasal regions. Histopathology..

[47] Yoon B-N, Chon K-M, Hong S-L (2013). Inflammation and apoptosis in malignant transformation of sinonasal inverted papilloma: the role of the bridge molecules, cyclooxygenase-2, and nuclear factor kappaB. Am J Otolaryngol.

[48] Zhang J, Yang Y, Tang Y, Wu X, Cong L, Ruan B (2014). The quantification and significance of muscle segment homeobox gene Msx2, human topoisomerase II-alpha, HPV16 and VEGF in sinonasal inverted papilloma. Lin Chuang Er Bi Yan Hou Tou Jing Wai Ke Za Zhi.

[49] Wu Q, Yang Y, Wu X (2012). Expression and significance of Msx2 and topo II-alpha in sinonasal inverted papilloma. Lin Chuang Er Bi Yan Hou Tou Jing Wai Ke Za Zhi.

[50] Wang Y, Ding L, Zhang X (2012). Clinical significance of programmed cell death 4 expression in malignant progression of human nasal inverted papillomas. Med Oncol.

[51] Yasukawa S, Kano S, Hatakeyama H, et al. Genetic mutation analysis of the malignant transformation of sinonasal inverted papilloma by targeted amplicon sequencing. Int J Clin Oncol. 2018;23(5):835–843. 10.1007/s10147-018-1296-1 29779136

[52] Zhang W, Wen S, Zhang T, Wang B, Gao W, Li L (2014). Expression and significance of PTEN and HIF-1alpha proteins in sinonasal inverted papilloma. Zhonghua Er Bi Yan Hou Tou Jing Wai Ke Za Zhi.

[53] Kakizaki T, Hatakeyama H, Nakamaru Y (2017). Role of microRNA-296-3p in the malignant transformation of sinonasal inverted papilloma. Oncol Lett.

[54] van Zijl FVWJ, Monserez DA, Korevaar TIM (2017). Postoperative value of serum squamous cell carcinoma antigen as a predictor of recurrence in sinonasal inverted papilloma. Clin Otolaryngol.

[55] Yasumatsu R, Nakashima T, Masuda M (2005). Clinical value of serum squamous cell carcinoma antigen in the management of sinonasal inverted papilloma. Head Neck..

[56] Yamashita Y, Uehara T, Hasegawa M (2016). Squamous cell carcinoma antigen as a diagnostic marker of nasal inverted papilloma. Am J Rhinol Allergy..

[57] Wang H-L, Lin Z-H, Fan G-K, Chen H-M (2007). Management of sinonasal inverted papilloma: endoscopic approach and lateral rhinotomy. Zhejiang da Xue Xue BaoYi Xue Ban.

[58] Cai Y, Zhang J (2012). Expression of fascin and correlation with MVD in sinonasal inverted papilloma. Lin Chuang Er Bi Yan Hou Tou Jing Wai Ke Za Zhi.

[59] Wu HH, Zafar S, Huan Y, Yee H, Chiriboga L, Wang BY (2009). Fascin over expression is associated with dysplastic changes in sinonasal inverted papillomas: a study of 47 cases. Head Neck Pathol..

[60] Marioni G, Brescia G, Nicole L (2018). Survivin and cortactin expression in sinonasal schneiderian (inverted) papilloma and associated carcinoma. Am J Rhinol Allergy..

[61] Suh JD, Palma-Diaz F, Bhuta S, Wang MB (2013). COX-(2) overexpression in sinonasal inverted papilloma. Int Forum Allergy Rhinol.

[62] Lee G-H, Yoon Y-H, Kim YM (2012). Pattern of expression of cyclooxygenase-2 in malignant transformation of sinonasal inverted papilloma. Am J Otolaryngol.

[63] Liu W, Li Z, Luo Q (2011). The elevated expression of osteopontin and vascular endothelial growth factor in sinonasal inverted papilloma and its relationship with clinical severity. Am J Rhinol Allergy..

[64] Wu Y, Cui S, Wu Q, Ma Z, Yuan W (2013). Expression and significance of osteopontin and muscle segment homeobox gene Msx2 in sinonasal inverted papilloma. Lin Chuang Er Bi Yan Hou Tou Jing Wai Ke Za Zhi.

[65] Stasikowska-Kanicka O, Wagrowska-Danilewicz M, Danilewicz M (2016). Immunohistochemical study EMT-related proteins in HPV-, and EBV-negative patients with Sinonasal Tumours. Pathol Oncol Res.

[66] Koo BS, Jung BJ, Kim SG, Liang ZL, Yeong MK, Rha KS (2011). Altered expression of E-cadherin and -catenin in malignant transformation of sinonasal inverted papillomas. Rhinology..

[67] Tsou Y-A, Huang H-J, Wang T-C, Tai C-J, Chen C-M, Chen CY-C (2014). Evaluation of correlation of cell cycle proteins and Ki-67 interaction in paranasal sinus inverted papilloma prognosis and squamous cell carcinoma transformation. Biomed Res Int.

[68] Sahnane N, Ottini G, Turri-Zanoni M (2019). Comprehensive analysis of HPV infection, EGFR exon 20 mutations and LINE1 hypomethylation as risk factors for malignant transformation of sinonasal-inverted papilloma to squamous cell carcinoma. Int J Cancer.

[69] Jalilvand S, Saidi M, Shoja Z, Ghavami N, Hamkar R (2016). The prevalence of human papillomavirus infection in Iranian patients with sinonasal inverted papilloma. J Chinese Med Assoc.

[70] Hoffmann M, Klose N, Gottschlich S (2006). Detection of human papillomavirus DNA in benign and malignant sinonasal neoplasms. Cancer Lett.

[71] McKay SP, Gregoire L, Lonardo F, Reidy P, Mathog RH, Lancaster WD (2005). Human papillomavirus (HPV) transcripts in malignant inverted papilloma are from integrated HPV DNA. Laryngoscope..

[72] Hasegawa M, Deng Z, Maeda H (2012). Human papillomavirus load and physical status in sinonasal inverted papilloma and squamous cell carcinoma. Rhinology..

[73] Mohajeri S, Lai C, Purgina B, et al. Human papillomavirus: an unlikely etiologic factor in sinonasal inverted papilloma. Laryngoscope. 2018;128(11):2443–2447. 10.1002/lary.27207 29668071

[74] Liu Y, Duan L, Tian J (2017). Role of the Akt/mTOR signaling pathway in human papillomavirus-associated nasal and sinonasal inverted papilloma. Acta Biochim Biophys Sin Shanghai.

[75] Zhong Z, Yan A, Jiang F, Wei H, Jiang X (2010). The study on the relationship between human papillomavirus infection and pathogenesis of nasal inverted papilloma and its malignant transformation. Lin Chuang Er Bi Yan Hou Tou Jing Wai Ke Za Zhi.

[76] Shen J, Tate JE, Crum CP, Goodman ML (1996). Prevalence of human papillomaviruses (HPV) in benign and malignant tumors of the upper respiratory tract. Mod Pathol.

[77] Yamashita Y, Hasegawa M, Deng Z (2015). Human papillomavirus infection and immunohistochemical expression of cell cycle proteins pRb, p53, and p16(INK4a) in sinonasal diseases. Infect Agents Cancer.

[78] Lin H, Lin D, Xiong X-S (2016). Roles of human papillomavirus infection and stathmin in the pathogenesis of sinonasal inverted papilloma. Head Neck.

[79] Kim J-Y, Yoon J-K, Citardi MJ, Batra PS, Roh H-J (2007). The prevalence of human papilloma virus infection in sinonasal inverted papilloma specimens classified by histological grade. Am J Rhinol.

[80] Udager AM, McHugh JB, Goudsmit CM (2018). Human papillomavirus (HPV) and somatic EGFR mutations are essential, mutually exclusive oncogenic mechanisms for inverted sinonasal papillomas and associated sinonasal squamous cell carcinomas. Ann Oncol.

[81] Rooper LM, Bishop JA, Westra WH (2017). Transcriptionally active high-risk human papillomavirus is not a common etiologic agent in the malignant transformation of inverted Schneiderian Papillomas. Head Neck Pathol..

[82] Bakhtin AA, Bykova VP, Daikhes NA, Karneeva OV (2018). Human papillomavirus and Epstein-Barr virus in the pathogenesis of inverted papilloma and associated sinonasal carcinoma. Arkh Patol.

[83] Zhao R-W, Guo Z-Q, Zhang R-X (2016). Human papillomavirus infection and the malignant transformation of sinonasal inverted papilloma: a meta-analysis. J Clin Virol.

[84] Govindaraj S, Wang H (2014). Does human papilloma virus play a role in sinonasal inverted papilloma?. Curr Opin Otolaryngol Head Neck Surg.

[85] Shojaku H, Fujisaka M, Yasumura S (2007). Positron emission tomography for predicting malignancy of sinonasal inverted papilloma. Clin Nucl Med.

[86] Kim JS, Kwon SH (2017). Different characteristics of a single sinonasal inverted papilloma from sequential PET-CT: a case report. Medicine (Baltimore).

